# Enhancing Skeletal Muscle Fiber Type Transition Through Substrate Coating Alteration in Myoblast Cell Culture

**DOI:** 10.3390/ijms26125637

**Published:** 2025-06-12

**Authors:** Yhusi Karina Riskawati, Chuang-Yu Lin, Akira Niwa, Hsi Chang

**Affiliations:** 1 International Ph.D. Program in Cell Therapy and Regenerative Medicine, College of Medicine, Taipei Medical University, Taipei 110301, Taiwan; 2 Department of Physiology, Faculty of Medicine, Universitas Brawijaya, Malang 65145, Indonesia; 3 Department of Biomedical Science and Environmental Biology, Kaohsiung Medical University, Kaohsiung 807378, Taiwan; 4 Department of Clinical Application, Center for iPS Cell Research and Application (CiRA), Kyoto University, Kyoto 606-8507, Japan; 5 Department of Pediatrics, School of Medicine, College of Medicine, Taipei Medical University, Taipei 110301, Taiwan; 6 Department of Pediatrics, Taipei Medical University Hospital, Taipei 110301, Taiwan

**Keywords:** Collagen I, Fibronectin, Geltrex™, C2C12 differentiation, muscle fiber type, transcriptomic

## Abstract

Skeletal muscle diseases often exhibit fiber-type-specific characteristics and pose substantial clinical challenges, necessitating innovative therapies. The extracellular matrix (ECM) plays a pivotal role in muscle physiology and regeneration, influencing cell differentiation. However, its specific role and mechanisms influencing muscle fiber type specification remain insufficiently understood. In this study, C2C12^GFP^ myoblasts were differentiated into myofibers on plates coated with fibronectin, Collagen I, and Geltrex™. Differentiation occurred successfully across all ECM substrates, resulting in myofiber formation. Quantitative polymerase chain reaction (qPCR) analysis confirmed myogenic marker expression patterns, indicating decreased *Pax7* and increased *Myog* levels by day 7. Protein analysis through Western blot and immunofluorescence assays along with transcriptomic profiling through RNA sequencing consistently indicated that Collagen I promoted slow-type fibers development, as evidenced by increased slow myofiber protein expression and the upregulation of slow fiber-associated genes, potentially mediated by pathways involving calcineurin/NFAT, MEF2, MYOD, AMPK, PI3K/AKT, and ERK1. In contrast, fibronectin and Geltrex™ led to fast-type fiber development, with elevated fast-type fiber protein levels and upregulation of fast fiber-associated genes, possibly through activation of HIF1A, FOXO1, NFKB, and ERK2. These findings elucidate ECM-mediated muscle fiber type differentiation mechanisms, informing future targeted therapies for muscle regeneration.

## 1. Introduction

Skeletal muscle diseases, including Duchenne muscular dystrophy (DMD), sarcopenia, and injuries resulting in volumetric muscle loss, often affect specific muscle fiber types [1], leading to impaired mobility and significant challenges in patient quality of life due to their progressive nature. Current treatments are primarily focused on managing symptoms rather than addressing the underlying causes of muscle degeneration [2], indicating a need for therapeutic strategies aimed at restoring muscle mass and function.

Leveraging the ECM, such as Collagen I and fibronectin, which plays a key role in muscle physiology and regeneration, has been identified as a promising approach to addressing these underlying causes of muscle degeneration [3,4,5]. The ECM provides essential structural support and regulates cellular functions, such as adhesion, proliferation, and differentiation in muscle tissue. Primarily composed of laminin, fibronectin, and collagen, the ECM is critical for maintaining muscle integrity and facilitating repair. Studies have indicated that ECM components influence muscle cell behavior, enhancing differentiation and tissue regeneration by replicating the natural cellular environment [6,7]. These findings highlight the potential of ECM-based therapies in improving outcomes in patients with muscle degenerative diseases and volumetric muscle loss injuries [5].

Studies have demonstrated that ECM coatings significantly influence muscle cell differentiation. For example, poly (L-lactic acid) (PLLA) fibers coated with ECM proteins were reported to enhance myoblast differentiation into myofibers, with cells expressing muscle markers such as myosin and alpha-actinin [8]. Additionally, muscle-derived ECM coatings on sinusoidal wavy surfaces have been demonstrated to promote myogenic differentiation by providing both biochemical cues and contact guidance [9,10]. Although the aforementioned studies have suggested that ECM coatings can replicate in vivo conditions to promote muscle differentiation, a gap remains in the understanding of the specific effects of various ECM coatings on muscle fiber type differentiation in vitro. Skeletal muscle is composed of two primary fiber types: fast-twitch (Type II) and slow-twitch (Type I). Type II fibers, characterized by rapid contraction and a high glycolytic capacity, are essential for rapid, powerful movements. By contrast, Type I fibers, which have high oxidative capacity, are crucial for sustained, repetitive activities [11].

In this study, we hypothesized that modifying ECM coatings would promote the differentiation of skeletal muscle fibers into specific types in C2C12 myoblast cultures. We systematically investigated the effects of various ECM coatings on myoblast differentiation to identify conditions that promoted targeted muscle fiber type specification. The findings could inform the development of therapeutic strategies for muscle regeneration.

The composition of the ECM plays a crucial role in muscle engineering and may influence fiber type specification. Collagen-type I hydrogels were among the earliest ECM components used in 3D-engineered muscle tissues, highlighting the importance of ECM composition in muscle engineering applications [12]. Lloyd et al. [13] revealed that the ECM composition varies between muscle fiber types, with slow-twitch fibers having higher collagen content than fast-twitch fibers. This variation underscores the potential influence of ECM composition on fiber type specification.

Duffy et al. [14] investigated the uniaxial alignment of human skeletal muscle cells on polydimethylsiloxane (PDMS) surfaces coated with various ECM proteins and revealed that Matrigel-coated surfaces yielded the most differentiated myotubes, although the researchers did not distinguish specific fiber types. Additionally, Ebrahimi et al. [15] demonstrated that collagen-coated dishes in a 3D culture model of DMD skeletal muscle could reverse the dystrophic phenotype, highlighting the relevance of ECM components in muscle disease modeling.

The present study focused on three specific ECM components: Collagen I, fibronectin, and Geltrex^TM^. Collagen I plays a fundamental role in muscle tissue engineering applications [12]. Han et al. [16] demonstrated that Fibronectin contains specific integrin-binding sites that modulate myoblast behavior. Geltrex™, a complex ECM mixture similar to Matrigel^®^, provides a diverse array of ECM components that may influence muscle cell differentiation and fiber type specification [14].

This study investigated the effects of various ECM components on the differentiation of C2C12^GFP^ myoblasts into fast- and slow-type muscle fibers. Experiments were conducted to analyze the temporal expression patterns of myogenic regulatory factors, fiber type-specific proteins, and gene expression profiles to identify ECM types that promote specific muscle fiber type transitions. The study findings help to provide a deeper understanding of the role of the ECM in muscle differentiation; the findings have potential applications for muscle regeneration and tissue engineering. This study offers insights that can help with treating conditions involving fast-type muscle degeneration, such as in DMD, sarcopenia-related atrophy, and fiber-type shift from oxidative to glycolytic in the lower limbs of patients with chronic obstructive pulmonary disease (COPD) [1,17].

## 2. Results

### 2.1. Collagen I, Fibronectin, and Geltrex™ Induced Myogenic Differentiation Comparable to the Control Condition

**Figure 1:**
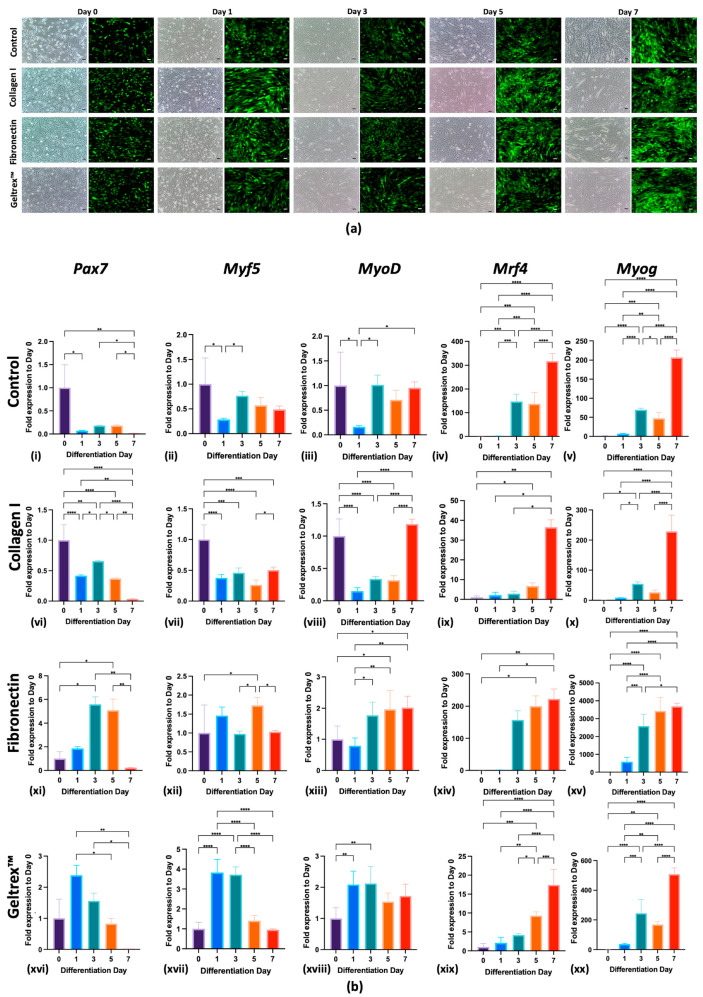
Differentiation and Myogenic Marker Expression of C2C12^GFP^ Cells on Various ECM Coatings. (**a**) C2C12^GFP^ Cell Differentiation Across Days 0, 1, 3, 5, and 7 for control, Collagen I, fibronectin, and Geltrex™ Coatings (scale bar = 100 μm). Sequential fluorescence and phase-contrast images depict the differentiation process from days 0 to 7. Green fluorescence indicates active myogenesis; phase-contrast images reveal morphology changes in cellular structure over time. (**b**) Temporal Expression Profiles of Myogenic Markers in C2C12^GFP^ Cells on control, Collagen I, fibronectin, and Geltrex™ Coatings. Expression levels of myogenic markers *Pax7*, *Myf5*, *MyoD*, *Mrf4*, and *Myog* in C2C12 cells are presented for days 0, 1, 3, 5, and 7 of differentiation (**b**(**i**–**xx**)). Each row presents data for a specific substrate coating: control, Collagen I, fibronectin, and Geltrex™. The columns present the expression of the aforementioned myogenic markers, normalized to day 0 levels. In all panels: * *p* < 0.05, ** *p* < 0.01, *** *p* < 0.001, **** *p* < 0.0001.

As indicated in Figure 1a, C2C12^GFP^ cells subjected to control, Collagen I, fibronectin, and Geltrex™ conditions were successfully differentiated by day 7. Under each condition, the cells exhibited early signs of elongation from day 3, with significant increases in density observed by days 5 and 7, as evidenced by strong GFP fluorescence, which indicates active myogenesis. These findings underscore the inherent capacity of C2C12^GFP^ cells to undergo myogenic differentiation leading to the formation of mature myofibers under all tested conditions.

These observations were further confirmed through qPCR analysis, which revealed that all substrates effectively supported the differentiation of C2C12^GFP^ cells, with differentiation comparable to that of the control condition. This was evidenced by the differential expression of key myogenic markers—*Pax7*, *Myf5*, *Myod*, *Mrf4*, and *Myog*—at specific differentiation time points (Figure 1b(i–xx)). As depicted in Figure 1b(i), the control condition facilitated myogenic differentiation, as evidenced by the upregulation of *Mrf4* and *Myog* and a decrease in *Pax7* by day 7. Additionally, Collagen I, fibronectin, and Geltrex™ enhanced the expression of these markers from the early stages of differentiation, shown in Figure 1b(vi,xi,xvi). By the end of the experiments, all conditions yielded a significant reduction in *Pax7*, indicating a transition from proliferation to differentiation, along with a marked increase in *Mrf4* and *Myog* levels, illustrated in Figure 1b(v,x,xv,xx), underscoring the maturation of myotubes into myofibers.

### 2.2. Collagen I Enhanced Slow Myofibers Protein Expression, Whereas Fibronectin and Geltrex™ Favored Fast Myofibers Protein Expression

**Figure 2:**
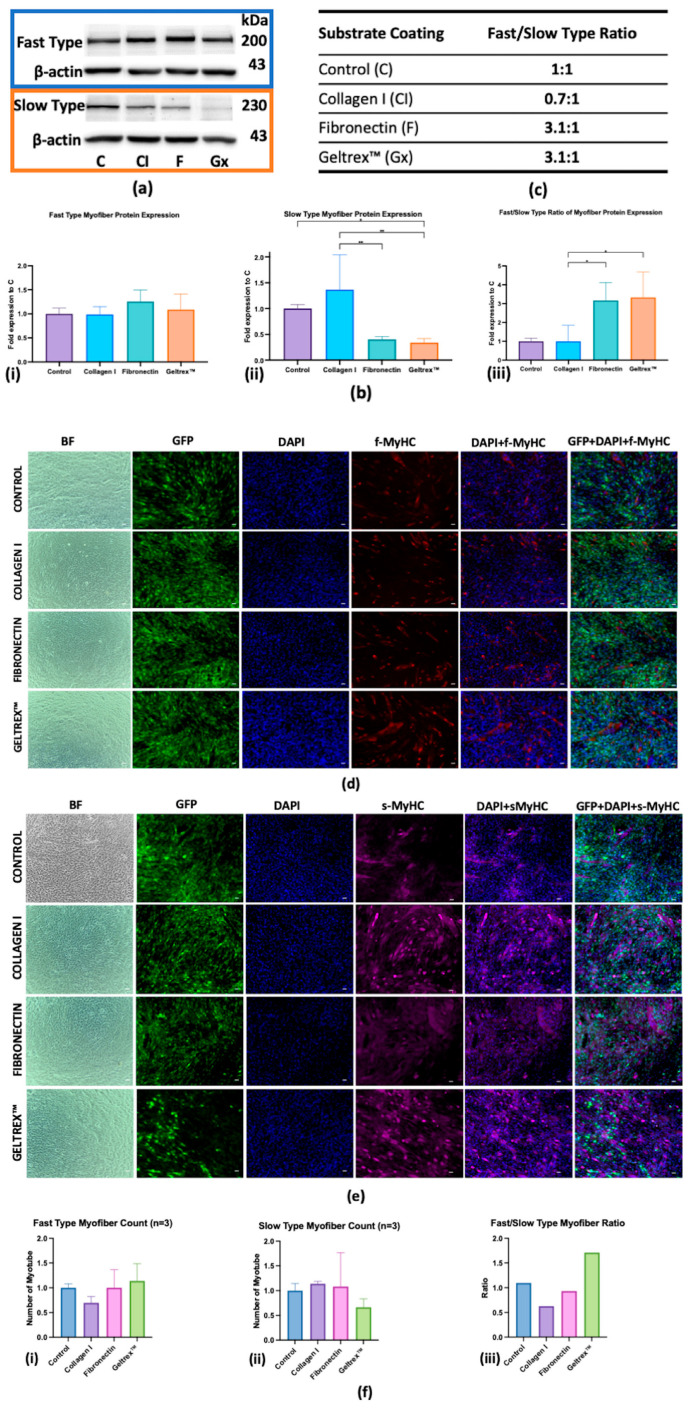
Myofiber Protein Expression in Response to Extracellular Matrix Coatings. (**a**) Western Blot Analysis: Protein levels of fast and slow isoforms across different ECM coatings across three replicates, with β-actin as the loading control. (**b**) Quantitative Analysis: Fold changes in the expression of protein isoforms for fast-type (left), slow-type (middle), and fast/slow ratio (right), with error bars indicating standard deviation (* *p* < 0.05, ** *p* < 0.01). (**c**) Fast/slow-type isoform expression ratios specific to each ECM coating. (**d**,**e**) Immunofluorescence characterization of fast and slow myosin heavy chain isoform expression in myofibers (scale bar = 100 μm). Panel (**d**) depicts the fast-type MyHC isoform (red), and panel (**e**) displays the slow-type MyHC isoform (magenta) with GFP localization, DAPI staining (blue nuclei), and merged images. (**f**) Quantitative assessment of fast-type (i), slow-type myofiber (ii) formation and ratio of fast-type and slow-type myofiber (iii).

Figure 2a–c depicts the protein expression dynamics of fast and slow myofiber types in C2C12 cells cultured on various extracellular matrix coatings—control, Collagen I, fibronectin, and Geltrex™—during the late stages of differentiation. A Western blot analysis revealed distinct expression patterns for both fast and slow myofiber protein types across these conditions. In the control condition, a balanced ratio of 1:1 was maintained between fast- and slow-type proteins. By contrast, under the Collagen I condition, a reduced ratio of 0.7:1 was noted, indicating a predominance of the expression of slow-type proteins. Fibronectin significantly favored the expression of fast-type proteins, resulting in a fast/slow ratio of 3:1. Similarly, Geltrex™ demonstrated a fast/slow ratio of 3.1:1, which promoted the differentiation of fast-type muscle fiber.

An immunofluorescence analysis (Figure 2d,e) was conducted to visualize the expression of MyHC isoforms in myofibers at advanced differentiation stages; the quantification is presented in Figure 2f. Fast MyHC expression was significantly higher in the Geltrex™ condition relative to the control condition, whereas a subtle decrease was observed in fibronectin, indicating a robust development of fast-twitch muscle fibers. By contrast, Collagen I demonstrated a less pronounced increase in fast MyHC expression. For slow MyHC expression, Collagen I markedly enhanced the myofibers count, thereby supporting the development of slow-twitch muscle fibers. This contrasted with the more moderate increases observed with fibronectin and the marked decrease observed with Geltrex™. The fast/slow MyHC ratio was the highest in the Geltrex™ condition, followed by the fibronectin condition, and the lowest ratio was observed in the Collagen I condition. Collectively, these findings indicate that both fibronectin and Geltrex™ favor the differentiation of fast-type myofibers.

### 2.3. Collagen I Enhanced Slow Fiber Type Gene Expression, Whereas Fibronectin and Geltrex™ Increased Fast Fiber Type Gene Expression

**Figure 3:**
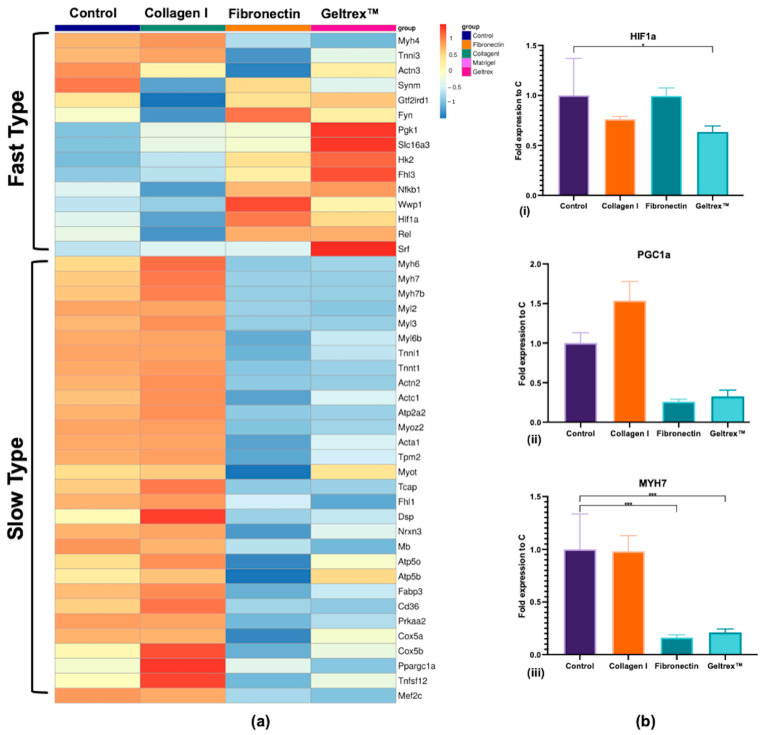
Gene Expression Profiles of Muscle Fiber Type Markers. (**a**) RNA-seq heatmaps presenting distinct gene expression profiles associated with fast-type and slow-type muscle fibers. Gene expression levels are color-coded, with blue indicating lower expression and red indicating higher expression, with a z-score close to 1. (**b**) qPCR analysis validating the RNA-seq findings for key muscle fiber type-related genes, specifically *Hif1a* (i), *Ppargc1a* (ii), and *Myh7* (iii) (* *p* < 0.05, *** *p* < 0.001).

An RNA-seq analysis was conducted that revealed 29,301 annotated transcripts. The expression patterns of muscle fiber type-specific genes, including contractile proteins and metabolic regulators, were compared across the control, Collagen I, fibronectin, and Geltrex™ conditions (Figure 3a). Key genes, such as *Hif1α*, *Pgc1α*, and *Myh7*, were validated through qPCR, and the results are presented in Figure 3b.

The expression levels of fast fiber-associated genes, such as *Fyn*, *Hk2*, *Synm*, and *Hif1α*, were increased in the fibronectin and Geltrex™ groups, whereas the expression levels of slow fiber-associated genes, such as *Myh7*, *Myh6*, *Myl2*, and *Ppargc1a*, were increased in the Collagen I group. Notably, the fast fiber genes were downregulated in the Collagen I condition, whereas the slow fiber genes were suppressed in the fibronectin and Geltrex™ conditions. However, qPCR revealed nuanced patterns for *Hif1α* which were not significantly elevated in fibronectin/Geltrex™, though still significantly suppressed in Collagen I. These collective findings indicate that Collagen I promotes slow fiber gene expression while concurrently reducing fast fiber expression whereas fibronectin and Geltrex™ favor the opposite effect, despite variation in individual gene responses.

### 2.4. Pathways and Key Regulators Potentially Involved in Slow Fiber Transition on the Collagen I Coating and Fast Fiber Transition on the Fibronectin and Geltrex™ Coatings

To elucidate the pathways responsible for the observed fiber type transitions, we analyzed the DEGs between the Collagen I, fibronectin, and Geltrex™ coatings. A DEG analysis was used to compare Collagen I with fibronectin and Geltrex™ to examine the transition to the slow fiber type associated with Collagen I. In addition, an analysis was conducted of the DEGs between fibronectin/Geltrex™ and Collagen I that was focused on the identification of pathways associated with the transition to the fast fiber type in the fibronectin and Geltrex™ conditions. A total of 1225 DEGs were identified in the comparison of Collagen I versus fibronectin, and 1343 DEGs were identified in the comparison between Collagen I versus Geltrex™.

**Figure 4:**
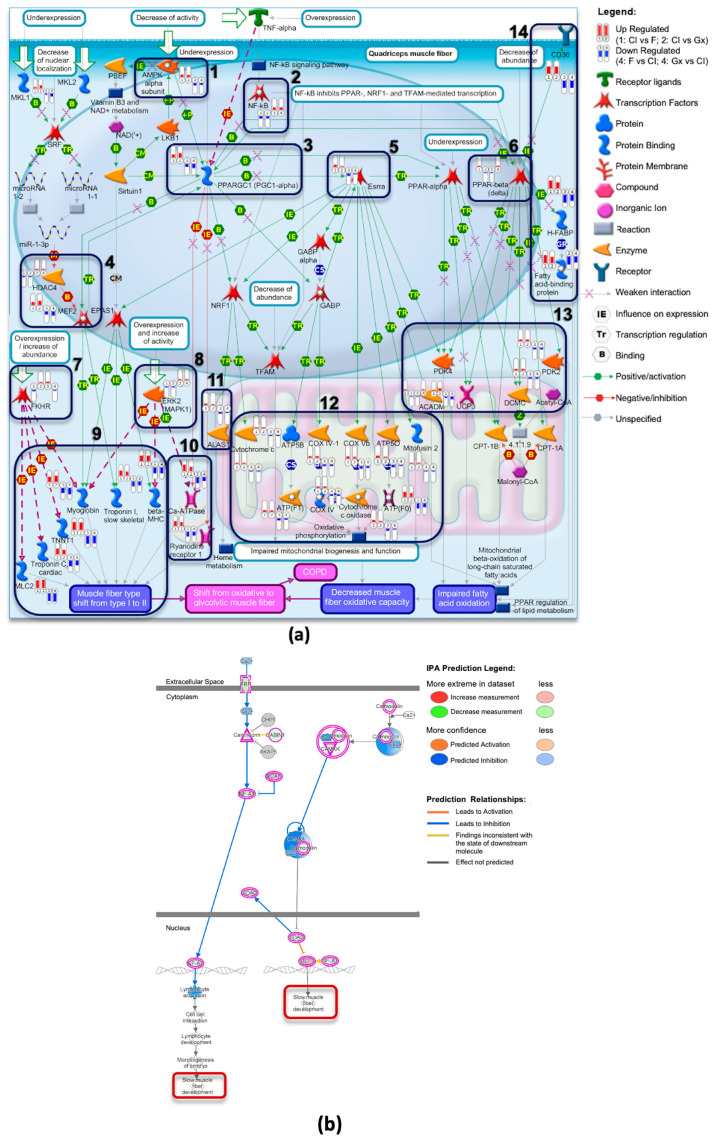
Pathway Analysis of Muscle Fiber Phenotype Shifts in Response to ECM Coatings. (**a**) Transition from oxidative to glycolytic muscle fiber phenotype in quadriceps muscles in patients with COPD. This complex pathway illustrates the molecular mechanisms underlying the transition from oxidative to glycolytic muscle fibers in patients with COPD. The capsule-shaped markers in the image represent gene expression levels, with red indicating upregulation and blue indicating downregulation. DEGs are grouped as follows: (1, 2) DEGs from Collagen I versus fibronectin and Geltrex™; (3, 4) DEGs from fibronectin and Geltrex™ versus Collagen I. (**b**) Selected region of the calcium signaling pathway from IPA analysis of DEGs (Collagen I vs. fibronectin). A focused view of the calcium signaling pathway derived from IPA analysis of the DEGs between Collagen I and fibronectin coatings, demonstrating how calcium ion (Ca2+) influx influences slow muscle fiber type development. Upregulated genes, highlighted in bright colors, include calmodulin, *CaMK*, *Mef2*, and *Nfat*, which promote slow muscle fiber development. Downregulated genes are indicated in blue. The arrows of varying colors, thicknesses, and line styles in the figure are explained in the MetaCore and IPA legends placed beside them.

MetaCore pathway analysis was employed to develop a specific pathway map, presented in Supplementary Figure S2 that indicated a transition from an oxidative to a glycolytic muscle fiber phenotype in the quadriceps muscles of patients with COPD. Notably, the gene expression data associated with Collagen I revealed an inverse pattern to this pathway, whereas the fibronectin and Geltrex™ conditions aligned with it, as displayed in Figure 4a. Specifically, markers for slow fiber types (*Myh7*, *Tnnt1*, *Tnni1*, *Tnnc1*, and *Myl2*) were upregulated in the Collagen I group but downregulated in the fibronectin and Geltrex™ groups. Additionally, genes related to oxygen transport (*Mb* and *Alas1*), intracellular calcium transport (*Ryr1* and *Atp2a1*), fatty acid binding and metabolism (*Cd36*, *Fabp3*, *Mydc*, and *Pdk2*), and mitochondrial function (*Cox family*, *Atp5b*, *Atp5o*, *Acadm*, and *Ucp3*) exhibited differential expression across the various coatings, as highlighted in Figure 4a (boxes 9–14). Additionally, regulatory elements exhibited opposing expression patterns (1–8 in Figure 4a), with AMPK subunits (*Prkaa2*), *Nfkb*, transcription factors (*Mef2*, *Foxo1*, and *Erk1/2*), and *Ppargc1a* as well as *Essra* and *Ppard* being upregulated in the Collagen I condition but downregulated in the fibronectin and Geltrex™ conditions. These findings are supported by significant GO terms, as presented in Supplementary Figure S3.

The IPA further supported these findings through the identification of significant canonical pathways, such as the calcium signaling and oxidative phosphorylation pathways, as depicted in Supplementary Figure S4. Notably, the calcium signaling pathway indicates the “slow fiber type development” function, which involves the upregulation of key proteins, such as calcineurin (*Ppp3ca*), calmodulin (*Calm1*), *CaMK*, and *Mef2*, and the downregulation of *Hdac* in the Collagen I condition, as presented in Figure 4b. Additionally, analyses regarding the KEGG pathway and GSEA curated pathway further corroborate these results, as indicated in Supplementary Figure S5.

**Figure 5:**
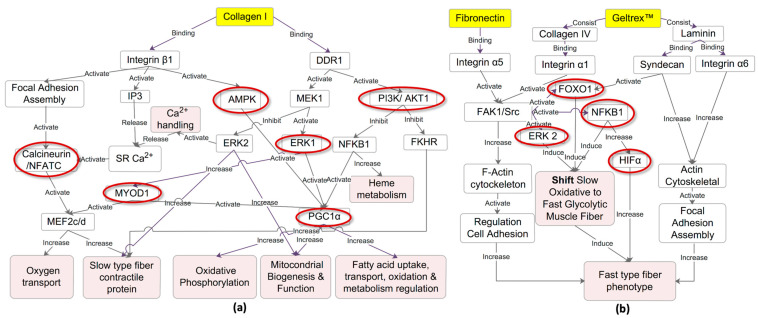
Predicted Regulatory Networks Influencing Muscle Fiber Type Transition by ECM Coatings. (**a**) Collagen I-induced slow fiber type transition: This network model illustrates how Collagen I binding to integrin β1 and DDR1 activates pathways such as calcineurin/NFATC, MEF2, MYOD, AMPK, PI3K/AKT, and ERK1. These pathways collectively drive mitochondrial biogenesis, oxidative phosphorylation, and upregulation of slow-type fiber contractile proteins. Inhibition of FOXO1 and NFKB1 further supports oxidative muscle characteristics. (**b**) fibronectin/Geltrex™-induced fast fiber type transition: This network depicts how fibronectin and Geltrex™ binding to integrins α5, α6, syndecan, and integrin α1 triggers FAK1/Src, ERK2, and HIFα signaling pathways, which promote actin cytoskeleton reorganization and focal adhesion assembly. Activation of HIF1A, FOXO1, NFKB1, and ERK2 facilitates the shift toward the glycolytic muscle fiber phenotype, characterized by the upregulation of fast-twitch fiber markers.

These findings strongly indicate that Collagen I coating promotes a transition toward an oxidative muscle fiber phenotype, whereas fibronectin and Geltrex™ coatings promote a shift toward a glycolytic phenotype. On the basis of the genes implicated in the enriched pathway analyses, the predicted upstream regulators were identified using IPA, and a reference was established regarding fiber type transition. The key upstream regulators selected for their relevance include PPP3CA, NFAT, MEF2, PPARGC1A, AMPK, ERK1/2, PI3K/AKT, MYOD1, HIF1A, FOXO1, and NFKB. The predicted network is presented in Figure 5a,b, with the network involving the calcineurin/NFAT, MEF2, MYOD, AMPK, PI3K/AKT, and ERK1 signaling pathways, which facilitate the transition to slow-type fibers on Collagen I. By contrast, the HIF1A, NFKB, FOXO1, and ERK2 signaling pathways promote the transition to fast-type fibers on fibronectin and Geltrex™.

## 3. Discussion

Numerous studies have leveraged the ECM for skeletal muscle regeneration, with various ECM components extensively used as coatings in two-dimensional cell culture systems and three-dimensional scaffolds for skeletal muscle tissue engineering [6,7]. Although these approaches have demonstrated potential in promoting myoblast differentiation, the specific effects of individual ECM components on muscle fiber type specification remain inadequately understood. The complex interactions between ECM composition and cellular signaling pathways have particularly increased the difficulty of identifying the precise mechanisms that govern fiber type specification in vitro. To address this challenge, we modified the conventional C2C12 myoblast culture system by introducing specific ECM coatings: Collagen I, fibronectin, and Geltrex™. These components were selected on the basis of their well-documented roles in muscle physiology and their previous applications in tissue engineering [12,14,15]. We hypothesized that these distinct ECM coatings would provide unique environments conducive to myoblast differentiation toward specific fiber types. We established an efficient system for investigating the effects of ECM composition on myogenic lineage determination, including the expression of fast and slow-type muscle fiber markers. Furthermore, we identified potential signaling pathways involved in ECM-mediated fiber type specification through transcriptomic analysis, thereby contributing to a deeper understanding of the role of the ECM in muscle differentiation.

During normal skeletal myogenesis, the initial wave of myogenic precursor cells in the dermomyotome expresses *Pax7*, followed by expression of *Myf5*. These sequential waves of myogenesis operate upstream of the primary myogenic transcription factor *Myod* and are further characterized by high expression levels of *Mrf4* and *Myog* during the late differentiation stage [18,19]. The findings of the present study indicate that all tested substrates effectively support myogenic differentiation. The differential expression of myogenic regulatory markers at various stages of myogenesis—specifically *Pax7*, which marks the transition of quiescent satellite cells (skeletal muscle stem cells) into proliferating myoblasts; *Myf5* and *Myod* during early differentiation when proliferative myoblasts commit to differentiation; and *Mrf4* and *Myog* during late differentiation as these cells fuse into myotubes and mature myofibers—indicates substrate-specific influences on the progression of myogenic differentiation, with differentiation comparable to that under control conditions [18]. These findings align with those of previous studies that have underscored the role of ECM components in promoting myogenic differentiation. The robust expression of myogenic markers such as *Pax7* on day 0, when the cells are still in a proliferative state, and their subsequent reduction by day 7 of differentiation, coupled with the increased expression of *Mrf4* and *Myog* across all substrates, further corroborate the supportive role of these substrates in muscle differentiation [20,21].

The extracellular matrix plays a crucial role in various aspects of skeletal muscle development, with its composition undergoing dynamic changes throughout the process of myogenesis [22]. The current findings of differential expression of fast and slow MyHC isoforms, as demonstrated in Western blot and immunofluorescence analyses in response to various ECM coatings, highlight the substrate-specific promotion of various muscle fiber types. Specifically, the preference of fibronectin and Geltrex™ for facilitating fast-twitch fiber differentiation contrasts with the slow-twitch-favoring environment provided by Collagen I [14,23]. These results elucidate the influence of the ECM microenvironment on muscle fiber type specification, suggesting a potential mechanism by which the developing muscle tissue’s surrounding matrix guides cellular fate decisions.

Our RNA-seq analysis revealed that Collagen I significantly enhanced the expression of genes associated with slow fiber types, whereas fibronectin and Geltrex™ promoted the expression of fast fiber type genes. Notably, nearly all slow fiber-associated genes, such as *Myh7* and *Myh6* (which are involved in slow-twitch muscle contraction) [24], *Myl2* (which regulates contractile force in slow fibers), as well as genes related to oxidative metabolisms such as the Cox family, Atpase family, and *Cd36* (fatty acid oxidase gene) [25], along with *Ppargc1a* (a master regulator of mitochondrial biogenesis) [26,27], were consistently upregulated in the presence of Collagen I. Conversely, exposure to fibronectin and Geltrex™ resulted in the upregulation of key fast fiber genes, including *Fyn* (which is crucial for fast-twitch growth and contraction) [28], *Hk2* (crucial for glycolytic metabolism) [25], *Synm* (which maintains the structural integrity of fast fibers) [29], *Hif1α* (which is involved in transition to fast-twitch muscle fibers) [24,30], and *Gtf2ird1* (which is the only gene in terms of GO related to the transition from slow to fast fiber types) [31].

The distinct gene expression profiles observed among the Collagen I, fibronectin, and Geltrex™ coatings underscore the pivotal role of the ECM in regulating transitions between muscle fiber types. However, the signaling pathways involved in these transitions remain underexplored. The complex interplay of signaling molecules and the cross-talk between various pathways, which are influenced by physiological stimuli, complicate the molecular mechanisms governing fiber type shifts [32,33,34]. Our IPA and MetaCore analysis, complemented by findings from GO, KEGG, and GSEA enrichment analyses, revealed significant signaling pathways. Collectively, these findings revealed that Collagen I promotes a transition toward slow oxidative fibers, whereas fibronectin and Geltrex™ favor the development of fast glycolytic fibers.

Central to the transition toward slow oxidative fibers is the upregulation of *Ppargc1a* observed in Collagen I [27,34]. In this study, increased *Ppargc1a* activity promoted the upregulation of mitochondrial genes essential for oxidative metabolism, a hallmark of slow-twitch muscle fibers. Notable examples of such genes include ATP synthase (*Atp5f1a*, *Atp5f1b*, and *Atp5po*) and cytochrome c oxidase (*Cox5b*, *Cox6a2*, and *Cox7a1*) subunits. Additionally, we observed an increased expression of genes involved in fatty acid metabolism, specifically *Cd36* and *Fabp3*, which facilitate fatty acid uptake and binding, as well as *Mydc* and *Pdk2*, which are crucial for metabolic regulation. The upregulation of oxygen transport-related genes (*Mb* and *Alas1*) further supports enhanced aerobic metabolism, a characteristic feature of slow-type fibers that contributes to their superior endurance levels [34]. This metabolic shift is accompanied by a notable upregulation of slow fiber type markers (*Myh7*, *Tnnt1*, *Tnni1*, *Tnnc1*, and *Myl2*), which are involved in slow-twitch muscle contraction and the regulation of contractile force in slow fibers. These expression patterns contrast with the shift from oxidative to glycolytic muscle fiber phenotypes observed in the quadriceps muscles of patients with COPD [32,35]. Conversely, fibronectin and Geltrex™ aligned with pathways where the oxidative metabolism characteristic of slow fibers transitions to glycolytic metabolism, which is typical of fast-type skeletal muscle fiber type, as observed in the quadriceps muscles of patients with COPD [17,25]. These findings have potential clinical implications: Collagen I-based approaches may be beneficial for muscle regeneration therapies aimed at addressing pathological fiber type shifts, such as those observed in the lower limbs of patients with COPD [36]. Conversely, fibronectin and Geltrex™ could prove valuable for studying DMD, where all fiber types undergo degeneration, but fast-twitch fibers are affected more rapidly. Additionally, these coatings may be relevant for addressing fast fiber type atrophy in sarcopenia and in developing disease models for drug screening aimed at preventing the degeneration or atrophy of specific muscle fiber types [37].

Our data revealed the differential regulation of key signaling components, including *Prkaa2* (AMPK), *Erk1/2*, *Pi3kca*, *Akt1*, *Nfkb*, *Foxo1*, *Myod*, and *Hif1a*, between the Collagen I and fibronectin/Geltrex™ conditions. These differences in signaling pathways may contribute to the promotion of either slow oxidative or fast glycolytic muscle fiber phenotypes. In the Collagen I coating, the upregulation of *Prkaa2* may be attributable to integrin binding creating an environment that favors slow-type myogenic differentiation by promoting cell adhesion and the activation of Ppargc1a signal transduction pathways [38,39]. Collagen I binding to the *Ddr1* receptor may lead to the activation of *Pi3kca*, *Akt1*, and subsequently *Erk1* and *Ppargc1a*, which are essential for the specific development of slow-type muscle fibers [23,33]. The involvement of MYOD in the development of both slow and fast fibers indicates a complex relationship between this transcription factor and fiber type specification. The findings regarding the regulation of MYOD, which was upregulated in the Collagen I coating, align with previous findings regarding its role in the transformation from fast to slow muscle fiber types [19,40].

The findings of overexpression of *Nfkb*, *Foxo1*, and *Erk2* in the fibronectin and Geltrex™ conditions may be attributable to their interaction with various receptors, such as integrins and syndecans, which activate FAK1/Src signaling pathways. This activation leads to a decrease in type I fibers through the downregulation of slow-type fiber genes such as *Myh7*, *Myl2*, *Tnnc1*, and *Tnnt1* along with a reduction in their oxidative capacity, facilitating a shift toward a fast fiber type phenotype [34,41]. Furthermore, the activation of FAK1/Src may promote remodeling of the F-actin cytoskeleton and regulate cell adhesion, ultimately contributing to the development of fast-type muscle fibers [42,43]. Additionally, the current study’s finding of upregulation of *Hif1a* in the fibronectin and Geltrex™ conditions aligns with its role in promoting glycolytic metabolism and characteristics associated with fast-twitch fibers [27,44].

The current study’s findings of differential activation of the calcium and calcineurin/NFAT signaling pathways between Collagen I and fibronectin/Geltrex™ coatings are particularly noteworthy. In the Collagen I condition, the upregulation of calcineurin (*Ppp3ca*), calmodulin (*Calm1*), and *CaMK*, along with the downregulation of *Hdac* and the upregulation of calcium-handling genes such as *Ryr1* and *Atp2a1*, may increase intracellular calcium release from the sarcoplasmic reticulum, thereby supporting the development of slow fiber types. This observation is consistent with the well-established belief regarding the role of calcium-dependent signaling in regulating muscle fiber type transitions during embryonic development and in adult muscle plasticity [19,35].

The findings of the intricate interplay between extracellular signals and intracellular pathways underscore the complex coordination involved in the determination of fiber type. Our predicted network results revealed the presence of complex cross-talk between various signaling pathways, as evidenced by interactions among calcium-dependent signaling molecules (e.g., *Nfatc, Mef2c, and Hdac4*), *Myod1*, and metabolic regulators (e.g., *Ppargc1a)*, as well as *Prkaa2*, *Pi3kca*, *Akt1* (PI3K/AKT), and *Erk1* in the Collagen I condition. By contrast, in the fibronectin/Geltrex™ conditions, interactions involving *Hif1a*, *Nfkb*, *Foxo1*, and *Erk2* were identified. These interactions indicate potential mechanisms underlying the fine-tuning of gene expression and maintenance of fiber type-specific gene programs. Moreover, they underscore the complex relationship between muscle activity, calcium handling, and metabolic adaptation in the determination of fiber type.

## 4. Materials and Methods

### 4.1. Cell Culture

C2C12^GFP^ myoblasts (Cellomics Technology, SC-102, Halethorpe, MD, USA) expressing green fluorescence protein (GFP) were cultured in high-glucose Dulbecco’s modified Eagle medium (DMEM) with L-glutamine (GIBCO, 12100-038, Paisley, Scotland, UK) supplemented with 10% fetal bovine serum (HyClone™, SH30070.03, Logan, UT, USA) and 1% penicillin-streptomycin (GIBCO, 15140-122, Paisley, Scotland, UK) at 37 °C in a 5% CO_2_ atmosphere. Cells were subcultured into multiwell plates (SPL Life Science, Pocheon-si, Gyeonggi-do, South Korea) once they reached 60–70% confluency. Differentiation was induced at 80–90% confluency by switching to a low-serum medium comprising 2% calf serum (HyClone™, SH30073.03, Logan, UT, USA), 1% nonessential amino acids (NEEA, 0823, ScienCell, Carlsbad, CA, USA), and 1% penicillin-streptomycin in DMEM. The medium was refreshed every 24–48 h [45].

### 4.2. Substrate Coating

Coating solutions were prepared in accordance with the manufacturers’ instructions. Collagen I (GEcoll, 110101, Hsinchu, Taiwan) was prepared in 0.01 N HCl (5 µg/cm^2^) and incubated for 1 h at room temperature. Fibronectin (Calbiochem, 4037443, Burlington, MA, USA) was dissolved in serum-free culture medium (5 µg/cm^2^) and applied for 45 min at room temperature. Geltrex™ LDEV-Free Reduced Growth Factor Basement Membrane Matrix (Fisher Scientific, A14132-01, Pittsburgh, PA, USA), which is primarily composed of laminin, Collagen IV, and entactin, was diluted to 1:100 in cold serum-free culture medium, applied as a thin gel, and incubated for 1 h at 37 °C. All coatings were rinsed with Dulbecco’s phosphate-buffered saline [D-PBS(-), 048-29805, FUJIFILM Wako, Saitama, Kawagoe, Japan] prior to cell seeding.

### 4.3. Quantitative Polymerase Chain Reaction Analysis

Total RNA isolation and complementary DNA (cDNA) synthesis were performed using commercial kits (Biotools Co., DPT-BD30, KRT-BA06-2, Kaohsiung, Taiwan) in accordance with the manufacturer’s instructions. Quantitative polymerase chain reaction (qPCR) was conducted using SYBR Green (Biotools Co., FPT-BB05-10, Kaohsiung, Taiwan) under the following amplification conditions: one cycle of 5 min at 95 °C, 40 cycles of 15 s at 95 °C, 30 s at 60 °C, and 30 s at 72 °C. The primer sequences used in this study are listed in Supplementary Table S1. Gene expression levels were analyzed using the ΔΔCT method.

### 4.4. Western Blot Analysis

Protein extraction and a Western blot analysis were performed as previously described [45]. Briefly, total protein was extracted on day 7 of differentiation by using a lysis buffer (Cell Signaling, 98035, Danvers, MA, USA), quantified using the Bradford protein assay (BioShop, BRA222.500, Burlington, ON, Canada), and separated using 8% sodium dodecyl sulfate-polyacrylamide gel electrophoresis (SDS-PAGE). The proteins were then transferred onto a polyvinylidene difluoride (PVDF) membrane (Millipore Immobilon-P, IPVH0016, Merck, Darmstadt, Germany) and probed with antibodies against fast-type myosin heavy chain (MyHC) isoforms (1:750 BS-5159R, Bioss Inc., Woburn, MA, USA), which were detected at 230 kDa, and slow-type MyHC (1:2500, GeneTex, NOQ7.5.4D, GTX11083, Irvine, CA, USA), with β-actin as the internal control. Band intensities were analyzed using ImageJ software version 1.52a and normalized to the internal control [46].

### 4.5. Immunofluorescence

The cultured cells were immunostained as previously described [40]. The cells were fixed with 4% paraformaldehyde for 15 min and permeabilized with 0.1% Triton X-100 for 10 min, after which they were blocked with 2% bovine serum albumin (BSA, ACE Biolabs, C30015, Taichung, Taiwan) for 10 min and mouse-to-mouse blocking solution (SkyTek Laboratories, AFI600, San Diego, CA, USA) for 1 h. Primary antibodies against fast MyHC (1:100, F59, DSHB, IA, USA) and slow MyHC (1:100, BA-D5, DSHB, IA, USA) were applied for 2 h. Alexa Fluor-conjugated secondary antibodies (488 and 549; Jackson ImmunoResearch Labs, 611-605-215, West Grove, PA, USA) were applied for 1 h. Nuclei were counterstained with 4′,6-diamidino-2-phenylindole (DAPI; 1:50). Images were captured using an ECHO Revolve Microscope at 10× magnification, with quantification performed using ImageJ on three randomly selected areas from three different wells per condition [46].

### 4.6. RNA Sequencing

RNA was extracted from triplicate samples for each group by following the procedures outlined in an earlier section (qPCR Analysis). RNA quality was assessed using a NanoDrop spectrophotometer (Thermo Fisher Scientific) to determine A260/A280 ratios (1.8–2.2), and RNA integrity was confirmed through gel electrophoresis. Library preparation and sequencing were performed using the Illumina NovaSeq 6000 platform with paired-end 150 bp reads. Initial data analysis, which included quality control (FastQC), read trimming (Trimmomatic), alignment (HISAT2), quantification (featureCounts), and differential expression analysis through relative log expression normalization by using DESeq2, was conducted using Biotools (Tursi Biotechnology Co., Taichung, Taiwan).

### 4.7. Biostatistics and Bioinformatics Analysis

Data from the qPCR, Western blot analysis, and immunocytochemical assay were analyzed using GraphPad Prism 8. Normality was assessed using the Shapiro–Wilk test, followed by one-way analysis of variance or the Kruskal–Wallis test, depending on the data distribution. Significant differences were further analyzed using Fisher’s least significant difference test or Dunn test (*p* < 0.05).

The variability and quality of the RNA sequencing data were assessed through principal component analysis (PCA) plots and Pearson’s correlation heat maps, generated using fragments per kilobase of transcript per million mapped reads (FPKM) with Biotools RNASeq v1.7.2, available online on https://cloud.toolsbiotech.com/ (accessed on 24 April 2024), as displayed in Supplementary Table S2 and Supplementary Figure S1.

Differentially expressed genes (DEGs) were further analyzed for gene ontology (GO) enrichment by using MetaCore, accessible via https://portal.genego.com/ (retrieved on 7 August 2024), with a threshold of 2 and *p*-adjusted value (*p*-adj) of ≤0.05. Pathway analyses were conducted using the Kyoto Encyclopedia of Genes and Genomes (KEGG; filtering DEGs for fold changes ≥ 2/−2, *p*-adj ≤ 0.05). Additionally, Gene Set Enrichment Analysis (GSEA) was performed with 1000 permutations (*p*-adj ≤ 0.25) by using curated pathway (Cu-P) gene sets from the Gene Set Knowledgebase (GSKB) through Biotools RNASeq v1.7.2. Additionally, ingenuity pathway analysis (IPA, Ingenuity System, available at www.ingenuity.com, last accessed on 31 December 2024) was conducted, with *p*-adj ≤ 0.05, and MetaCore was employed for comprehensive pathway analysis, including upstream regulator and downstream target analyses.

## 5. Conclusions

In conclusion, our findings revealed that all tested ECM substrate coatings—Collagen I, fibronectin, and Geltrex™—promoted myogenic differentiation of C2C12^GFP^ cells comparable with a control group. Notably, Collagen I significantly enhanced the expression of proteins and genes with the slow fiber type, suggesting a shift toward oxidative or slow-twitch fiber differentiation. Conversely, fibronectin and Geltrex™ promoted the expression of fast fiber proteins and associated genes, fostering glycolytic, fast-type fibers. A complex interplay of signaling pathways and transcriptional regulators was observed in this study. Specifically, Collagen I promoted slow-twitch fiber characteristics through the activation of the calcineurin/NFAT signaling pathway as well as the MYOD, AMPK, PI3K/AKT, and ERK1 signaling pathways. Fibronectin and Geltrex™ were demonstrated to enhance fast-twitch fiber traits through the HIF1A, NFKB, FOXO1, and ERK2 signaling pathways. These findings not only contribute to a deeper understanding of muscle plasticity but also suggest directions for the development of targeted therapeutic interventions.

While the present study provided crucial insights, it has some limitations that should be considered. This study was conducted entirely in vitro, using C2C12 myoblasts; therefore, the findings may not fully replicate the in vivo skeletal muscle environment. Although such a model can be valuable for mechanistic studies, in vivo experiments must be conducted to validate the study findings. Additionally, long-term studies are required to assess the stability and functionality of the differentiated muscle fibers over time. Furthermore, although the gene expression profiles observed in this study are informative, they may not be directly correlated with protein function. Future research should incorporate proteomic analyses and consider using primary human myoblasts to enhance the relevance of the findings to human muscle physiology.

## Data Availability

The processed data, metadata, and raw Illumina FASTQ files have been deposited in the National Center for Biotechnology Information Gene Expression Omnibus (GEO Accession number: GSE279968). Other data that support the findings of this study are available on request from the corresponding author.

## Supplementary Materials

The following supporting information can be downloaded at: https://www.mdpi.com/article/10.3390/ijms26125637/s1.

## Abbreviations

The following abbreviations are used in this manuscript:

AMPK	AMP (Adenosine monophosphate)-activated protein kinase
C2C12^GFP^	C2C12 (Immortalized mouse myoblast cell line) cells expressing green fluorescent protein
COPD	Chronic obstructive pulmonary disease
DEG	Differentially expressed gene
DMD	Duchenne muscular dystrophy
DSHB	Developmental Studies Hybridoma Bank
ERK	Extracellular signal-regulated kinase
FOXO1	Forkhead box O1
GO	Gene Ontology
GSEA	Gene Set Enrichment Analysis
GSKB	Gene Set Knowledge Base
HIF	Hypoxia-inducible factor
IPA	Ingenuity Pathway Analysis
KEGG	Kyoto Encyclopedia of Genes and Genomes
MEF2	Myocyte enhancer factor 2
MYOD	Myogenic differentiation 1 (a muscle-specific transcription factor)
NFATC	Nuclear factor of activated T-cells, cytoplasmic (specific isoform)
NFKB1	Nuclear factor kappa-light-chain-enhancer of activated B cells 1
PCA	Principal component analysis
PI3K	Phosphoinositide 3-kinase

## References

[1] Talbot J., Maves L. (2016). Skeletal muscle fiber type: Using insights from muscle developmental biology to dissect targets for susceptibility and resistance to muscle disease. Wiley Interdiscip. Rev. Dev. Biol..

[2] Tabebordbar M., Wang E.T., Wagers A.J. (2013). Skeletal Muscle Degenerative Diseases and Strategies for Therapeutic Muscle Repair. Annu. Rev. Pathol. Mech. Dis..

[3] Ahn J., Jeong H.E., Kim J., Chung S. (2020). Engineering hydrogel ECM scaffold component for in vitro 3D skeletal muscle tissue. Proceedings of the 21st International Conference on Miniaturized Systems for Chemistry and Life Sciences MicroTAS 2017.

[4] Lee H., Ju Y.M., Kim I., Elsangeedy E., Lee J.H., Yoo J.J., Atala A., Lee S.J. (2020). A novel decellularized skeletal muscle-derived ECM scaffolding system for in situ muscle regeneration. Methods.

[5] Ge X., Jin Y., He J., Jia Z., Liu Y., Xu Y. (2025). Extracellular matrix in skeletal muscle injury and atrophy: Mechanisms and therapeutic implications. J. Orthop. Transl..

[6] Schüler S.C., Liu Y., Dumontier S., Grandbois M., Le Moal E., Cornelison D., Bentzinger C.F. (2022). Extracellular matrix: Brick and mortar in the skeletal muscle stem cell niche. Front. Cell Dev. Biol..

[7] Ahmad K., Shaikh S., Chun H.J., Ali S., Lim J.H., Ahmad S.S., Lee E.J., Choi I. (2023). Extracellular matrix: The critical contributor to skeletal muscle regeneration-a comprehensive review. Inflamm. Regen..

[8] Fujita H., Shimizu K., Nagamori E. (2009). Novel method for fabrication of skeletal muscle construct from the C2C12 myoblast cell line using serum-free medium AIM-V. Biotechnol. Bioeng..

[9] Choi Y.-J., Park S.J., Yi H.-G., Lee H., Kim D.S., Cho D.-W. (2018). Muscle-derived extracellular matrix on sinusoidal wavy surfaces synergistically promotes myogenic differentiation and maturation. J. Mater. Chem. B.

[10] Chan A.H.P., Jain I., Oropeza B.P., Zhou T., Nelsen B., Geisse N.A., Huang N.F. (2023). Combinatorial extracellular matrix cues with mechanical strain induce differential effects on myogenesis in vitro. Biomater. Sci..

[11] Greising S.M., Gransee H.M., Mantilla C.B., Sieck G.C. (2012). Systems Biology of Skeletal Muscle: Fiber Type as an Organizing Principle. Wiley Interdiscip. Rev. Syst. Biol. Med..

[12] Khodabukus A. (2021). Tissue-Engineered Skeletal Muscle Models to Study Muscle Function, Plasticity, and Disease. Front. Physiol..

[13] Lloyd E.M., Pinniger G.J., Murphy R.M., Grounds M.D. (2023). Slow or Fast: Implications of Myofiber Type and Associated Differences for Manifestation of Neuromuscular Disorders. Acta Physiol..

[14] Duffy R.M., Sun Y., Feinberg A.W. (2016). Understanding the Role of ECM Protein Composition and Geometric Micropatterning for Engineering Human Skeletal Muscle. Ann. Biomed. Eng..

[15] Ebrahimi M., Lad H., Fusto A., Tiper Y., Datye A., Nguyen C.T., Jacques E., Moyle L.A., Nguyen T., Musgrave B. (2021). De novo revertant fiber formation and therapy testing in a 3D culture model of Duchenne muscular dystrophy skeletal muscle. Acta Biomater..

[16] Han W.M., Jang Y.C., García A.J., Wagner W.R., Sakiyama-Elbert S.E., Zhang G., Yaszemski M.J. (2020). 2.1.5—The Extracellular Matrix and Cell–Biomaterial Interactions. Biomaterials Science.

[17] Iepsen U.W., Pedersen B.K. (2020). Development of Limb Muscle Dysfunction in Chronic Obstructive Pulmonary Disease: Smoking, Inflammation, or Simply Disuse?. Am. J. Respir. Cell Mol. Biol..

[18] Asfour H.A., Allouh M.Z., Said R.S. (2018). Myogenic regulatory factors: The orchestrators of myogenesis after 30 years of discovery. Exp. Biol. Med..

[19] Lu T., Zhu Y., Guo J., Mo Z., Zhou Q., Hu C.Y., Wang C. (2023). MDFI regulates fast-to-slow muscle fiber type transformation via the calcium signaling pathway. Biochem. Biophys. Res. Commun..

[20] Dessauge F., Schleder C., Perruchot M.-H., Rouger K. (2021). 3D in Vitro Models of Skeletal Muscle: Myopshere, Myobundle and Bioprinted Muscle Construct. Vet. Res..

[21] Sun M., Chi G., Li P., Lv S., Xu J., Xu Z., Xia Y., Tan Y., Xu J., Li L. (2018). Effects of Matrix Stiffness on the Morphology, Adhesion, Proliferation and Osteogenic Differentiation of Mesenchymal Stem Cells. Int. J. Med. Sci..

[22] Thorsteinsdóttir S., Deries M., Cachaço A.S., Bajanca F. (2011). The extracellular matrix dimension of skeletal muscle development. Dev. Biol..

[23] Goetsch K.P., Kallmeyer K., Niesler C.U. (2011). Decorin Modulates Collagen I-Stimulated, but Not Fibronectin-Stimulated, Migration of C2C12 Myoblasts. Matrix Biol..

[24] Lin C.Y., Niwa A., Hou C.Y., Tsai C.M., Chang H. (2020). Bidirectional myofiber transition through altering the photobiomodulation condition. J. Photochem. Photobiol. B.

[25] Lynch C.J., Xu Y., Hajnal A., Salzberg A.C., Kawasawa Y.I. (2015). RNA sequencing reveals a slow to fast muscle fiber type transition after olanzapine infusion in rats. PLoS ONE.

[26] Albuquerque A., Óvilo C., Núñez Y., Benítez R., López-Garcia A., García F., Félix M.D.R., Laranjo M., Charneca R., Martins J.M. (2021). Transcriptomic Profiling of Skeletal Muscle Reveals Candidate Genes Influencing Muscle Growth and Associated Lipid Composition in Portuguese Local Pig Breeds. Animals.

[27] Rasbach K.A., Gupta R.K., Ruas J.L., Wu J., Naseri E., Estall J.L., Spiegelman B.M. (2010). PGC-1α regulates a HIF2α-dependent switch in skeletal muscle fiber types. Proc. Natl. Acad. Sci. USA.

[28] Yamada E., Bastie C.C., Koga H., Wang Y., Cuervo A.M., Pessin J.E. (2012). Mouse skeletal muscle fiber-type-specific macroautophagy and muscle wasting are regulated by a Fyn/STAT3/Vps34 signaling pathway. Cell Rep..

[29] García-Pelagio K.P., Muriel J., O’Neill A., Desmond P.F., Lovering R.M., Lund L., Bond M., Bloch R.J. (2015). Myopathic changes in murine skeletal muscle lacking synemin. Am. J. Physiol. Cell Physiol..

[30] Lunde I.G., Anton S.L., Bruusgaard J.C., Rana Z.A., Ellefsen S., Gundersen K. (2011). Hypoxia inducible factor 1 links fast-patterned muscle activity and fast muscle phenotype in rats. J. Physiol..

[31] Palmer S.J., Taylor K.M., Santucci N., Widagdo J., Chan Y.K., Yeo J.L., Adams M., Gunning P.W., Hardeman E.C. (2012). GTF2IRD2 from the Williams-Beuren critical region encodes a mobile-element-derived fusion protein that antagonizes the action of its related family members. J. Cell Sci..

[32] Delling U., Tureckova J., Lim H.W., De Windt L.J., Rotwein P., Molkentin J.D. (2000). A calcineurin-NFATc3-dependent pathway regulates skeletal muscle differentiation and slow myosin heavy-chain expression. Mol. Cell. Biol..

[33] Zhang S., Chen X., Huang Z., Chen D., Yu B., Chen H., He J., Luo J., Zheng P., Yu J. (2019). Leucine promotes porcine myofibre type transformation from fast-twitch to slow-twitch through the protein kinase B (Akt)/forkhead box 1 signalling pathway and microRNA-27a. Br. J. Nutr..

[34] Wang Y., Pessin J.E. (2013). Mechanisms for fiber-type specificity of skeletal muscle atrophy. Curr. Opin. Clin. Nutr. Metab. Care.

[35] Tu M.K., Levin J.B., Hamilton A.M., Borodinsky L.N. (2016). Calcium signaling in skeletal muscle development, maintenance and regeneration. Cell Calcium.

[36] Mou K., Chan S.M.H., Vlahos R. (2024). Musculoskeletal crosstalk in chronic obstructive pulmonary disease and comorbidities: Emerging roles and therapeutic potentials. Pharmacol. Ther..

[37] Fernández-Costa J.M., Fernández-Garibay X., Velasco-Mallorquí F., Ramón-Azcón J. (2021). Bioengineered in vitro skeletal muscles as new tools for muscular dystrophies preclinical studies. J. Tissue Eng..

[38] Grover C.N., Gwynne J.H., Pugh N., Hamaia S., Farndale R.W., Best S.M., Cameron R.E. (2012). Crosslinking and Composition Influence the Surface Properties, Mechanical Stiffness and Cell Reactivity of Collagen-Based Films. Acta Biomater..

[39] Liu H., Niu A., Chen S.E., Li Y.P. (2011). Beta3-integrin mediates satellite cell differentiation in regenerating mouse muscle. Faseb J..

[40] Huang B., Jiao Y., Zhu Y., Ning Z., Ye Z., Li Q.X., Hu C., Wang C. (2021). Mdfi Promotes C2C12 Cell Differentiation and Positively Modulates Fast-to-Slow-Twitch Muscle Fiber Transformation. Front. Cell Dev. Biol..

[41] Mittal A., Bhatnagar S., Kumar A., Lach-Trifilieff E., Wauters S., Li H., Makonchuk D.Y., Glass D.J., Kumar A. (2010). The TWEAK–Fn14 system is a critical regulator of denervation-induced skeletal muscle atrophy in mice. J. Cell Biol..

[42] Osório Alves J., Matta Pereira L., Cabral Coutinho do Rêgo Monteiro I., Pontes dos Santos L.H., Soares Marreiros Ferraz A., Carneiro Loureiro A.C., Calado Lima C., Leal-Cardoso J.H., Pires Carvalho D., Soares Fortunato R. (2020). Strenuous Acute Exercise Induces Slow and Fast Twitch-Dependent NADPH Oxidase Expression in Rat Skeletal Muscle. Antioxidants.

[43] Shiwarski D.J., Tashman J.W., Tsamis A., Bliley J.M., Blundon M.A., Aranda-Michel E., Jallerat Q., Szymanski J.M., McCartney B.M., Feinberg A.W. (2020). Fibronectin-based nanomechanical biosensors to map 3D surface strains in live cells and tissue. Nat. Commun..

[44] Chaillou T. (2018). Skeletal Muscle Fiber Type in Hypoxia: Adaptation to High-Altitude Exposure and Under Conditions of Pathological Hypoxia. Front. Physiol..

[45] Koma R., Shibaguchi T., Araiso Y., Yamada T., Nonaka Y., Jue T., Masuda K. (2023). TOM complex-independent transport pathway of myoglobin into mitochondria in C2C12 myotubes. Physiol. Rep..

[46] Wayne R. ImageJ. https://imagej.net/ij/.

