# Supplementary material for: Enhancing Skeletal Muscle Fiber Type Transition Through Substrate Coating Alteration in Myoblast Cell Culture
Source: Int J Mol Sci. 2025 Jun 12;26(12):5637. doi: 10.3390/ijms26125637 (PMC12192587; doi:10.3390/ijms26125637)
Supplement: Supplementary file 1 [file ijms-26-05637-s001.zip › ijms-3655286-supplementary.pdf]

Supplementary Figures and Tables

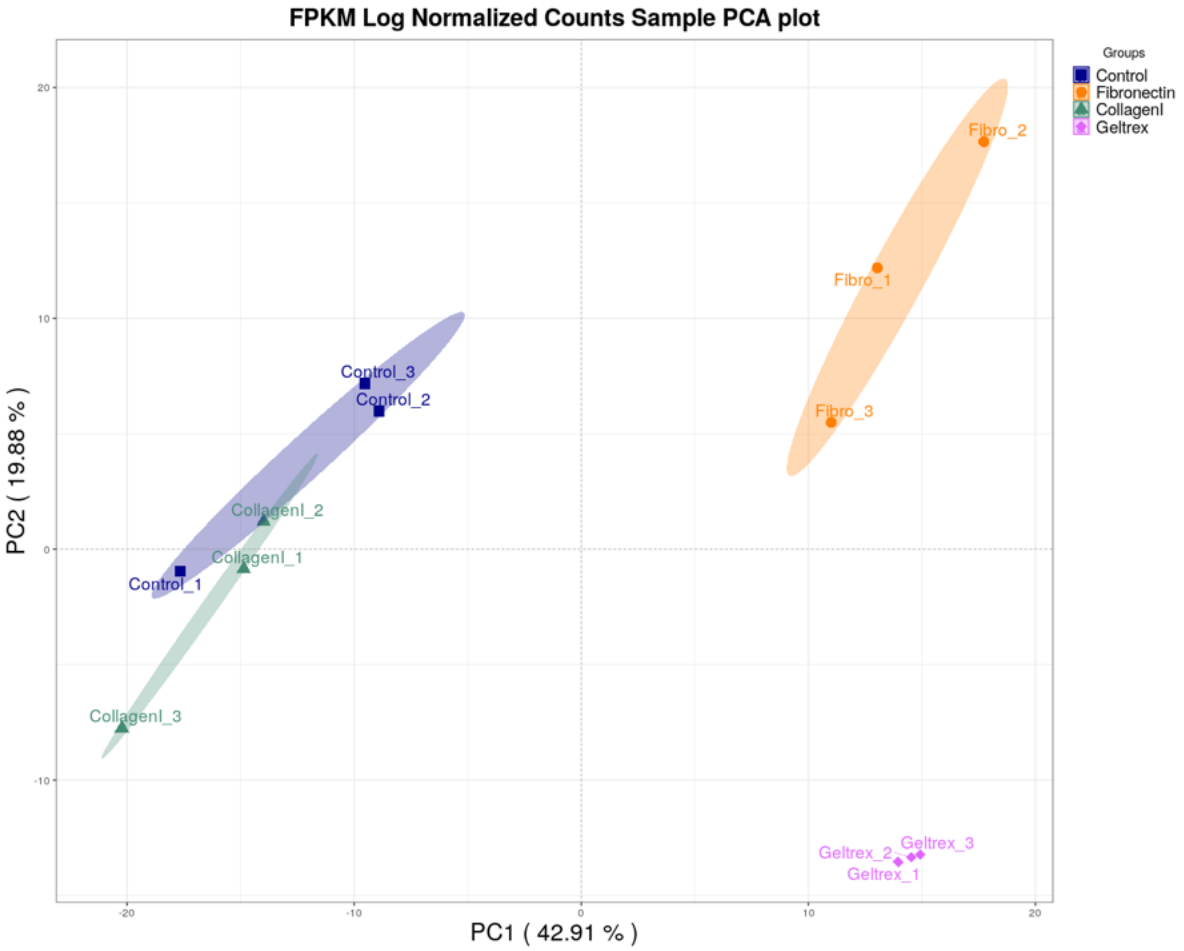

Figure S1. Sample Clustering by ECM Coating via Principal Component Analysis (PCA). This PCA plot shows the 6 clustering of replicate RNA-seq samples based on gene expression profiles. Each point represents a sample, and 7 the colors indicate the ECM coating condition: Control (purple), Collagen I (green), Fibronectin (orange), and 8 Geltrex™ (pink). The distinct clustering indicates consistent grouping of samples according to coating type.

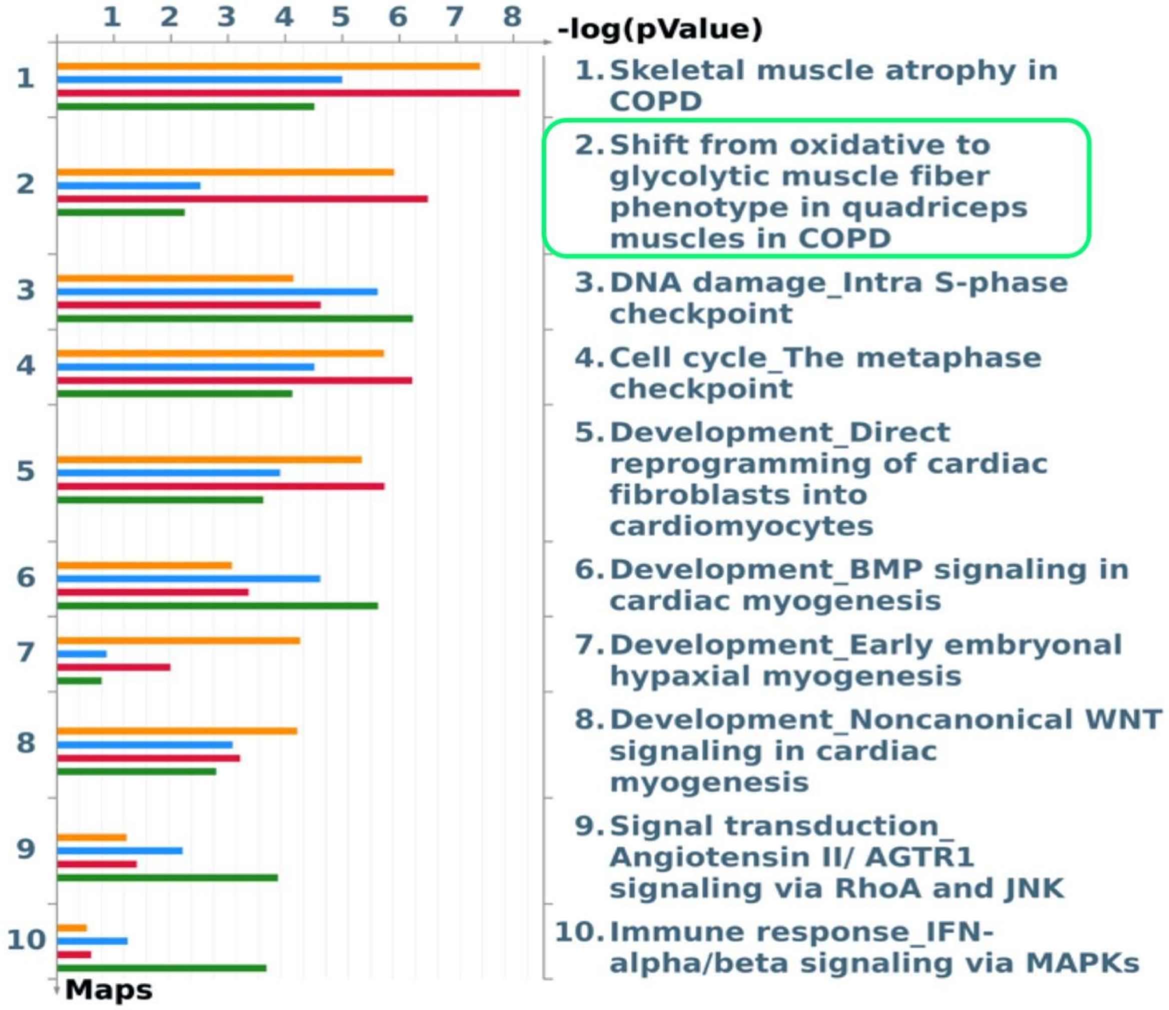

Figure S2. MetaCore Pathway Map of Differentially Expressed Genes (DEGs). The MetaCore pathway map presents significantly enriched pathways identified through MetaCore analysis based on a threshold log2fold change of 2 and *p*-adjusted value of 0.05. Pathways are listed on the vertical axis, with the log2fold change on the horizontal axis. DEGs are color-coded by comparison group, with orange for Collagen I vs. Fibronectin, blue for Collagen I vs. Geltrex™, red for Fibronectin vs. Collagen I, and green for Geltrex™ vs. Collagen I. The green box highlights the relevant pathway enrichment maps.

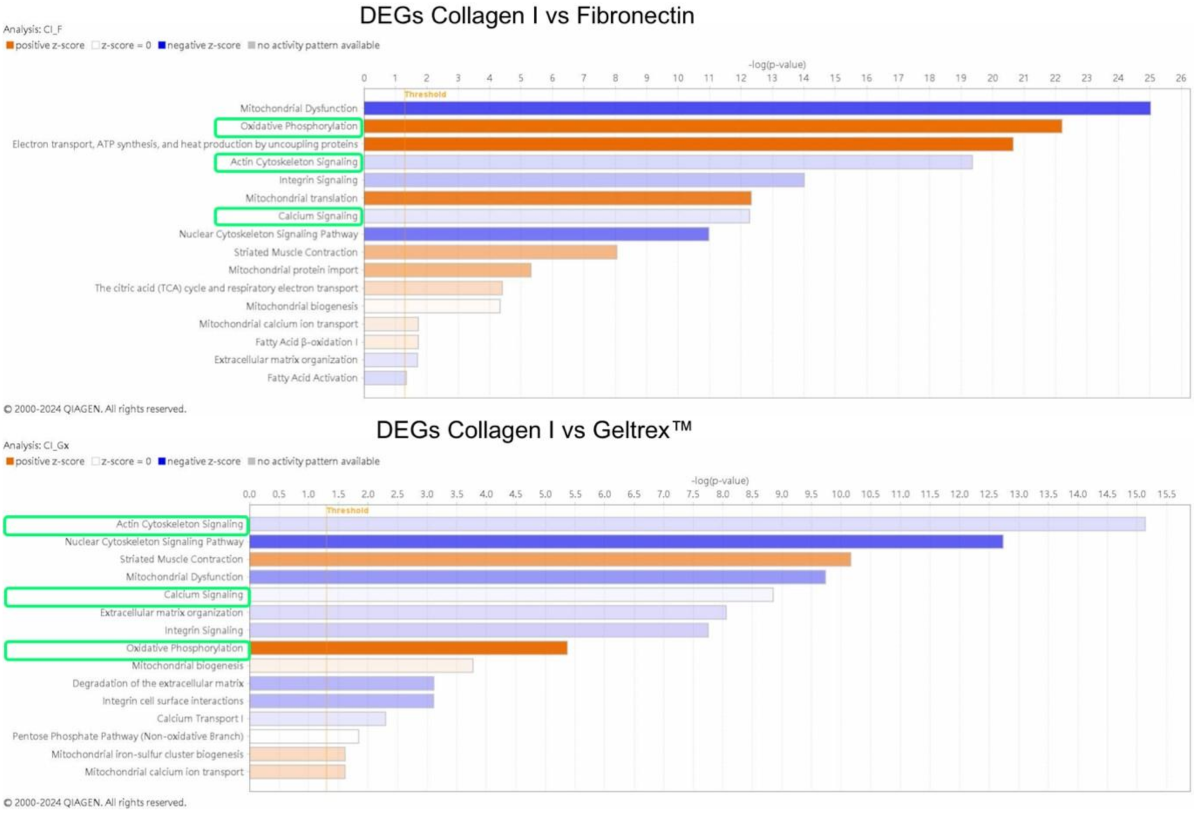

Figure S3. IPA Canonical Pathway Analysis. The figure presents the top significantly enriched canonical pathways related to skeletal muscle transition, as identified using ingenuity pathway analysis (IPA) based on log ratio expression with an adjusted *p* value of 0.05. The x-axis presents the log ratio expression, whereas the y-axis presents the pathways relevant to the experimental findings. Pathways are color-coded on the basis of z-score activation from blue to red (−1 to 1). The green boxes highlight the most relevant canonical pathways with significant enrichment.

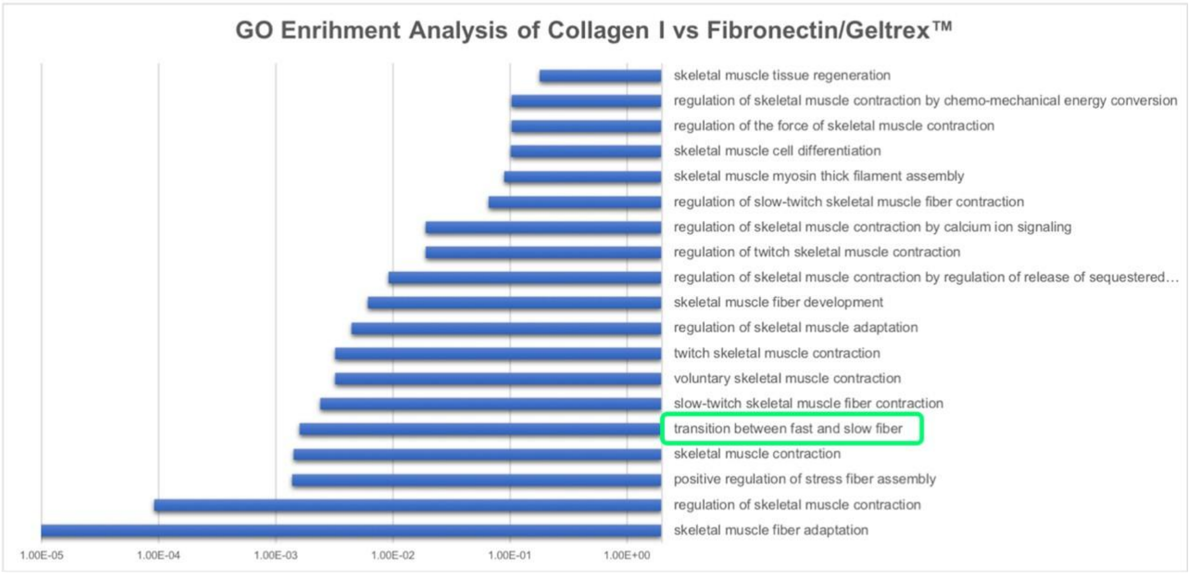

Figure S4. Selected Relevant GO Terms from MetaCore Enrichment Analysis of DEGs in Collagen I vs. Fibronectin/Geltrex™. This figure presents key biological processes related to skeletal muscle function and regulation, as identified using the

MetaCore analysis. The x-axis presents the false discovery rates for each selected GO term, whereas the y-axis presents the biological processes deemed most relevant to the experimental context.

The green box highlights the GO terms with the most significant enrichment.

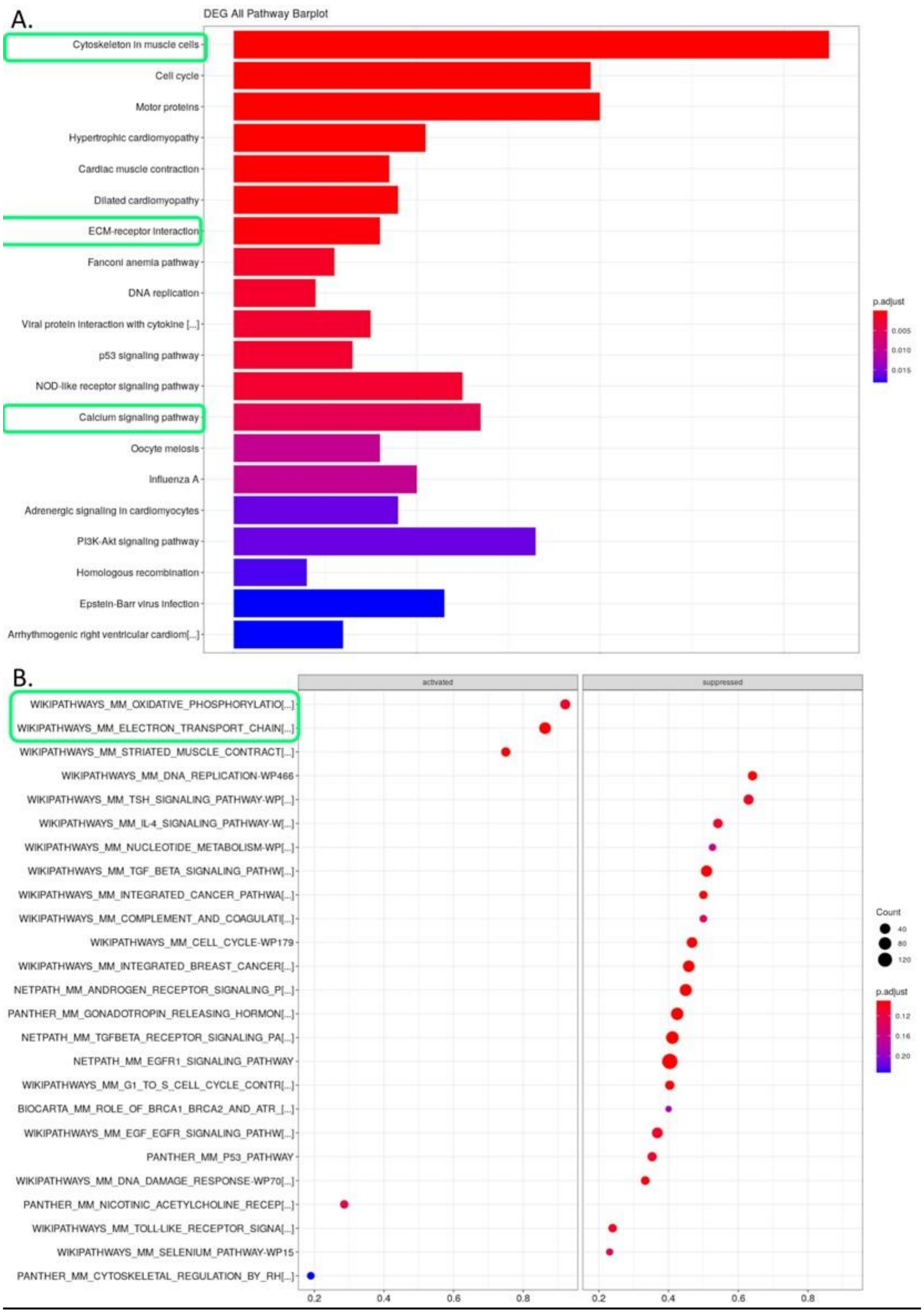

Figure S5. KEGG Pathway Analysis and GSEA GSKB Curated Pathway Analysis. **A.** Comparison of pathway enrichment across the top 10 significantly enriched KEGG pathways Collagen I, vs. Geltrex™ coatings, presented as −log (*p* value). The x-axis presents the gene count, and the y-axis presents the pathways, color-coded by significance levels. The green boxes highlight the pathways with the most relevant enrichment for each coating condition. **B.** The top significantly enriched pathways from the GSEA GSKB curated dataset. The x-axis presents the gene ratio, whereas the y-axis presents the gene sets, with color coding indicating significance. The size of the data points corresponds to the number of core enrichment genes within each pathway. The green boxes highlight the pathways that exhibit the most relevant enrichment.

Table S1. Primers Employed in qPCR in this Study

Gene	Forward primer sequence (5'-3')	Reversed primer sequence (5'-3')
Actb	GTGGAGATTGTTGCCATCAACGA	TACTCCTTGGAGGCCATGTAG
Pax7	GACTCGGCTTCCTCCATCTCAGCCA	CTG GCT GTA CTG GTA GC
Myf5	GCCTGCAAAGCTTGCAAGAGGA	TAAGTAACTGTCAATTAATTGCCTC
Myod	GATATGGAGCTTCTATCGCCGCCAC	TGGATCTAGGACAGACTTCTGCTC
Myf6 (Mrf4)	CTATTTCTTCTACTTAGATGGAGAA	ATT TGG TCC TGG CAA GGG
Myog (Myogenin)	ACTACCTTCCTGTCCACCTTCAG	ACC TTA CAT GCC CTC GGC AGG C
Hif1a	GTTTACTAAAGGACAAGTCACC	TTCTGTTTGAAGGGAG
Myh7	TACAATGCGCAAGTGGTAGC	GACGGTCTTACCAGCTCCG
Ppargc1a	AGGTCCCCAGGCAGTAGAT	CGTGCTCATTGGCTTCATA

Table S2. Quality Control of RNA Sequencing Results

Sample	Raw Read	Raw Base	Clean Read	Clean base	Q20 (%)	Q30 (%)	GC content (%)
Control_1	67082010	10.1 G	59299352	8.9 G	99.495	97.815	46.68
Control_2	73179112	11.1 G	64806918	9.7 G	99.505	97.85	46.78
Control_3	66685794	10.1 G	58305228	8.7 G	99.505	97.88	45.68
CollagenI_1	63814472	9.6 G	55322776	8.3 G	99.495	97.795	46.61
CollagenI_2	72300322	10.9 G	63364056	9.5 G	99.5	97.845	46.33
CollagenI_3	68249500	10.3 G	59545298	8.9 G	99.475	97.745	46.17
Fibro_1	75440302	11.4 G	66014736	9.9 G	99.5	97.825	46.87
Fibro_2	67636554	10.2 G	59312962	8.9 G	99.525	97.945	45.69
Fibro_3	70473300	10.6 G	62366664	9.3 G	99.525	97.93	46.64
Geltrex_1	65835272	9.9 G	56985844	8.5 G	99.49	97.785	47
Geltrex_2	68379704	10.3 G	59228890	8.8 G	99.5	97.85	47.02
Geltrex_3	63522700	9.6 G	54746972	8.2 G	99.48	97.765	46.8

Q=Quartile; Fibro: Fibronectin
